# Case Report: Efficacy of efgartigimod for autonomic dysfunction in patients with autoimmune glial fibrillary acidic protein astrocytopathy: a case series of 3 patients

**DOI:** 10.3389/fimmu.2026.1882447

**Published:** 2026-08-31

**Authors:** Huayi Ma, Bingying Xie, Yi Wu, Houshi Zhou, Feiyu Ma, Chuming Huang, Xiujun Zheng

**Affiliations:** 1 Department of Neurology, Shantou Central Hospital, Shantou, China; 2 Department of Nephrology, Shantou Central Hospital, Shantou, China

**Keywords:** autonomic neurological dysfunction, case series, efgartigimod, GFAP-A, urinary and defecation disorders

## Abstract

**Introduction:**

Efgartigimod can rapidly reduce serum IgG antibody levels and decrease antibody-mediated neuroinflammation and damage. It is primarily used to treat autoimmune diseases. Glial fibrillary acidic protein astrocytopathy (GFAP-A) is a disease of the central nervous system characterized by GFAP antibodies. It has a high incidence of autonomic dysfunction in patients and there is currently no effective treatment plan. It is not known whether efgartigimod can improve autonomic dysfunction in GFAP-A.

**Case presentation:**

All three patients with GFAP-A presented with autonomic dysfunction, which manifested as urinary and defecatory disorders. Marked remission of corticosteroid-refractory autonomic dysfunction was observed following combined treatment with efgartigimod and corticosteroids. All three patients presented with autonomic dysfunction. Patient 1 experienced marked improvement in autonomic function after the addition of efgartigimod, despite showing no significant improvement after high-dose pulse steroid therapy. Patient 2 showed no significant improvement after treatment with low-dose steroids, but achieved complete recovery of autonomic dysfunction after efgartigimod treatment. Patient 3 initially received low-dose steroids, with no significant improvement in autonomic dysfunction. After switching to a high dose of steroids combined with efgartigimod, the patient’s autonomic dysfunction gradually improved.

**Conclusion:**

We observed that after combined treatment with efgartigimod and corticosteroids, the corticosteroid-refractory autonomic dysfunction in GFAP-A patients was significantly alleviated, providing a new clinical treatment option. However, this article is based on a small sample size and requires further validation through larger-scale studies.

## Introduction

Glial fibrillary acidic protein astrocytopathy (GFAP-A) is a rare autoimmune disease of the central nervous system that was first identified in 2016 (1). The pathogenesis of this disease is unclear, but it primarily involves an abnormal immune response to GFAP autoantibodies. The inflammatory pathway involves both non-specific and specific immunity, abnormal activation of antigen-specific CD8^+^ T cells is a key upstream driver of GFAP-IgG antibody production. GFAP (glial fibrillary acidic protein) is an intracellular protein in astrocytes, so under normal conditions, antibodies cannot directly bind to it. However, when the body experiences infections (e.g., viral) or has an underlying tumor, immune activation may occur through molecular mimicry or paraneoplastic immune responses. This can lead to the generation of GFAP-specific CD8^+^ T cells. Upon recognizing and attacking astrocytes that express GFAP, these activated CD8^+^ T cells release cytokines (such as interferon-γ and tumor necrosis factor-α) and secrete granzymes, resulting in astrocyte damage and the subsequent release of intracellular GFAP. The released antigens further stimulate B cells, ultimately driving the production of GFAP-IgG antibody. The GFAP-IgG antibody serves as a highly specific diagnostic biomarker (2–5). The clinical manifestations of GFAP-A are broad and heterogeneous, with the most common presentation being meningoencephalitis, with or without myelitis. Other symptoms include headaches, psychiatric symptoms, extrapyramidal syndromes and autonomic dysfunction. The most common autonomic dysfunctions in GFAP-A patients are bladder dysfunction, gastrointestinal symptoms, as well as heart rate variability, blood pressure variability, and abnormal sweating (6). Currently, there is no unified treatment guideline for GFAP-A. First-line treatments typically include high-dose corticosteroids, intravenous immunoglobulin (IVIG) and plasma exchange (PE). The majority of patients have a monophasic disease course and respond well to corticosteroids (7). However, some patients with high antibody titers, extensive lesions, or comorbid tumors are resistant to steroids or experience relapse, requiring second-line therapies such as plasma exchange, immunoglobulin, or immunosuppressants (8).

Efgartigimod is a human IgG monoclonal antibody fragment which binds to the Fc region of IgG, thereby promoting its circulation or degradation via the neonatal Fc receptor (FcRn) (9, 10). It shows promise in the treatment of autoimmune diseases, such as myasthenia gravis (MG) (11, 12). The ADAPT trial (13) was a multicenter, randomized, double-blind, placebo-controlled Phase 3 study that enrolled 167 patients with generalized myasthenia gravis (gMG), of whom 129 were AChR-Ab positive. The results showed that the efgartigimod treatment group had significant improvements in both the Myasthenia Gravis Activities of Daily Living (MG-ADL) score and the Quantitative Myasthenia Gravis (QMG) score, with a safety profile comparable to placebo. Another study (14) involving 30 patients with seronegative MG (negative results for AChRAb and muscle-specific tyrosine kinase antibodies) demonstrated that efgartigimod could rapidly improve clinical symptoms, with the MG-ADL score significantly decreasing, some patients achieving Minimal Symptom Expression (MSE) as early as the first treatment cycle, and the overall safety was good. This confirms that its efficacy is not limited to the AChR antibody-positive population. Furthermore, results from a Phase 2 clinical trial (15) of subcutaneous efgartigimod in adults with chronic inflammatory demyelinating polyradiculoneuropathy (CIDP) indicated that the drug has a favorable safety and tolerability profile in CIDP patients, can quickly improve clinical symptoms, and significantly reduces the risk of disease relapse. There are also case reports (16) showing that efgartigimod is highly effective in GFAP-A patients who are insensitive to steroid treatment, leading to marked improvements in both clinical symptoms and brain lesions. The incidence of autonomic dysfunction in GFAP-A patients ranges from 20% to 40%, and autonomic dysfunction persisting after corticosteroid therapy is a common and disabling sequela (17). However, the effect of efgartigimod in improving autonomic function in patients with persistent autonomic dysfunction after hormone therapy has not yet been clearly mentioned. Here, we observed that after combined treatment with efgartigimod and corticosteroids, the corticosteroid-refractory autonomic dysfunction in GFAP-A patients was significantly alleviated.

## Case presentation

### Case 1

A 65-year-old male patient was admitted on 5 October 2024, due to a fever and cough accompanied by an altered mental state and difficulty urinating and defecating.

The neurological physical examination revealed restlessness and incoherent speech. The patient was uncooperative during the examination. There was voluntary movement of all limbs and neck stiffness. The submental-thoracic distance was three fingerbreadths. There was a negative Kernig’s sign and a negative bilateral Babinski sign.

Further relevant examinations showed low serum sodium at 122 mmol/L (reference 137–147 mmol/L), high immunoglobulin E at 333.7 IU/mL (reference 10–90 IU/mL), and elevated glycated hemoglobin at 6.6% (reference 4%–6%). Serum IgG was 10.9 g/L, within the normal range. A routine blood test and tests for hypersensitive C-reactive protein, procalcitonin, respiratory virus nucleic acid, dengue antigen, liver function, tumour markers, the T-Sport test and an infectious disease panel showed no significant abnormalities. Chest CT scan revealed mild pneumonia. Lower abdominal CT scan revealed prostatic calcification.

Cerebral MRI (plain and enhanced) showed scattered lacunar infarcts with no abnormal inflammatory lesions. Plain MR scans of the cervical, thoracic and lumbar spine show degenerative changes, with no obvious spinal cord compression. The cerebrospinal fluid (CSF) examination revealed the following: pressure of 150 mm H2O (reference range: 80–180 mm H2O); white blood cell count of 22×10^6/L (reference range: 0-8×10^6/L); glucose level of 4.04 mmol/L (reference range: 2.8-4.5 mmol/L); chloride level of 123.5 mmol/L (reference range: 120–132 mmol/L); and IgG content of 0.151 g/L (reference range: 0-0.1 g/L). CSF protein quantification was 1.56 g/L (reference range: 0.15-0.45 g/L). CSF microbiological examination was negative.

After anti-infection treatment, the patient’s fever subsided on the 7th day of admission. However, restlessness, incoherent speech and significant abdominal distension persisted, accompanied by urinary and defecatory dysfunction. Continuous bladder catheterization, gastrointestinal decompression and manual rectal evacuation were required. Low-dose corticosteroid therapy (10 mg dexamethasone for four days) was initiated, leading to a slight improvement in restlessness; however, urinary and defecatory dysfunction, as well as incoherent speech, remained prominent. The Clinical Assessment Scale for Autoimmune Encephalitis (CASE) score was 10 (with memory impairment accounting for 2 points, psychiatric symptoms for 2 points, language dysfunction for 3 points and gait instability for 3 points), the modified Rankin Scale (mRS) score was 4 and the bladder and rectal function scores were 4 and 3 respectively [as evaluated by the Expanded Disability Status Scale (EDSS)].

Immunological results: Indirect immunofluorescence assay of cells detected positive GFAP antibody IgG in CSF (1:32) and serum (1:10). Anti-AQP4 IgG, anti-MOG IgG and other autoantibodies against autoimmune encephalitis were not detected in CSF or serum.

Based on the patient’s clinical manifestations, cerebrospinal fluid findings, and neurological examination, the diagnosis was GFAP-A. Due to the patient’s financial constraints leading to refusal of intravenous IgG and the absence of plasmapheresis facilities at our hospital, after full communication, on the 11th day of admission, we administered intravenous efgartigimod at a dose of 1200 mg, combined with intravenous methylprednisolone pulse therapy (1 g for three days). The patient’s symptoms of irritability and incoherent speech disappeared, their abdomen became less distended, and their urination and defecation functions improved. The serum IgG level decreased to 5.73 g/L. On the 15th day of admission, the same dose of efgartigimod was administered intravenously again. Upon discharge, the patient could urinate and defecate independently, with a CASE score of 1 (gait instability: 1 point), an mRS score of 1, and a bladder and rectal function score of 1 each. A follow-up CSF examination 1 month later showed a positive result for the GFAP antibody (1:1). Mycophenolate mofetil tablets (0.5g twice daily) were initiated as oral immunosuppressive therapy at discharge and maintained for four months before reducing to 0.25 g twice daily, and discontinued after eight months. After discharge, the patient received methylprednisolone at a dose of 40 mg per day, reducing by 5 mg every two weeks until reaching a dose of 10 mg per day, which was maintained for three months before reducing to 5 mg per day. This was discontinued after six months. Follow-up cranial MRI scans showed no significant changes compared to previous scans.

### Case 2

A 21-year-old male patient presented chills and fever in early August 2025. After taking cephalosporin medication, his temperature normalized, followed by difficulty defecating, urinary frequency, urgency and incontinence, accompanied by dizziness, slowed reactions and slurred speech. His condition deteriorated, leading to hospitalization. Upon admission, physical examination revealed slightly slowed reactions and slurred speech. Both pupils were equal in size and round, with brisk light reflexes, and the eyeballs moved freely, with no nystagmus. Both nasolabial grooves were symmetrical and the tongue was in the midline. Muscle strength was grade 5- in all four limbs, with tremors in both upper limbs and increased muscle tone in all four limbs. Bilateral sensory function was symmetrical. There were hyperactive deep tendon reflexes in both upper limbs and active deep tendon reflexes in both lower limbs. Bilateral coordination was accurate. The patient also exhibited neck stiffness and bilateral Kernig’s signs (+), with negative bilateral Babinski signs.

Relevant examinations after admission were as follows: complement C3 level: 0.663 g/L (reference range: 0.79–1.52 g/L); complement C4 level: 0.155 g/L (reference range: 0.16–0.38 g/L); serum sodium concentration; routine blood test; routine urine test; high-sensitivity C-reactive protein (hs-CRP); liver function; metabolism; rheumatology panel; antinuclear antibody spectrum; thyroid function; T-Sport test; HIV; and non-specific syphilis antibody. None of these tests revealed any significant abnormalities. Color Doppler ultrasonography of the urinary system showed no significant abnormalities. Lower abdominal CT indicated bladder wall thickening.

After admission, the patient received treatment for infection and viruses, as well as a low dose of the dexamethasone (10 mg for two days). The patient still exhibited a slow response and slurred speech, accompanied by urinary and fecal incontinence. The CASE score was 4 (memory dysfunction: 1 point; language dysfunction: 1 point; gait instability: 1 point; fatigue: 1 point), the mRS score was 2, the bladder function score was 2, and the rectal function score was 2.

**Figure 1:**
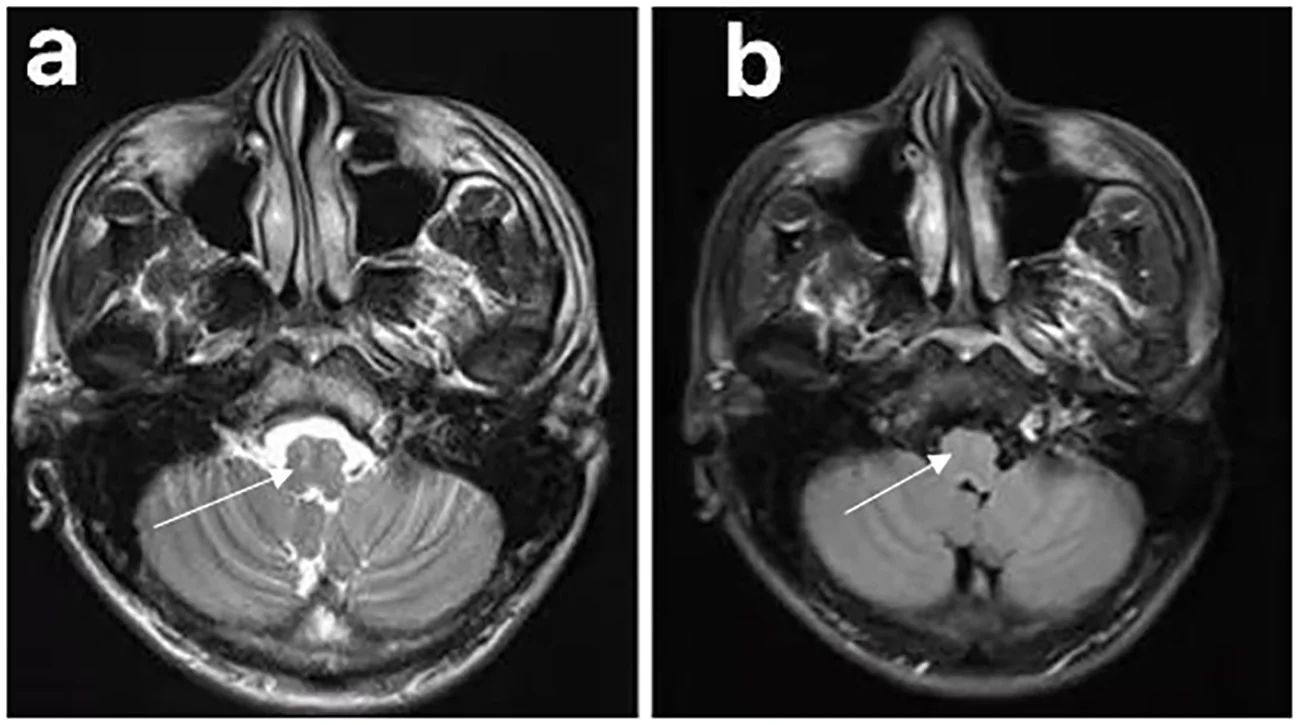
Brain magnetic resonance imaging (MRI) findings of patient 2. **(A)** T2-weighted imaging shows hyperintensity in the medulla oblongata. **(B)** T2-FLAIR imaging shows mild hyperintensity in the same region, with indistinct margins. No obvious abnormal enhancement is observed on contrast-enhanced scanning.

A cranial MRI scan (plain and enhanced) revealed a suspicious lesion in the medulla oblongata, indicating the possibility of inflammation (**Figure 1**). Electroencephalogram: Abnormal EEG (mildly slowed background activity, amplitude difference between corresponding left and right regions less than 50%, and occipital rhythmic assimilation to photic stimulation). CSF examination revealed an opening pressure of 157 mmH2O (reference range: 80–180 mmH2O), a white blood cell count of 64×10^6/L (reference range: 0-8×10^6/L), glucose 2.5 mmol/L (reference range: 2.8-4.5 mmol/L), chloride 126.2 mmol/L (reference range: 120–132 mmol/L), and CSF protein 0.36 g/L (reference range: 0.15-0.45 g/L). Pathogen and microbial detection in the CSF were negative.

Immunological findings: An indirect immunofluorescence assay of the CSF detected GFAP antibody IgG positivity (1:32), while other autoimmune encephalitis antibodies were not detected. Serum autoimmune encephalitis antibodies were negative.

Based on the new examinations and tests, the patient was diagnosed with GFAP-A. The patient refused high-dose corticosteroid therapy and intravenous immunoglobulin (IgG). After full communication, on the 4th day of admission, we administered intravenous efgartigimod at a dose of 1200 mg, combined with dexamethasone (20 mg for 5 days) intravenously. Upon discharge, the patient occasionally experienced mild dizziness. The slow response and slurred speech symptoms had disappeared. Limb muscle strength was grade 5 and defecation and urination functions were normal. The CASE score was 0, the mRS score was 1, the bladder function score was 0 and the rectal function score was 0. After discharge, the patient was prescribed prednisone tablets at 50 mg per day, with a weekly reduction of 5 mg. Mycophenolate mofetil tablets (0.5 g twice daily) were also initiated as oral immunosuppressive therapy. The patient was followed up for four months after discharge without relapse.

### Case 3

A 59-year-old male patient presented with a fever, cough, sputum production and general malaise on 21 February 2026. He was admitted to a tertiary medical center in China on 26 February 2026. Laboratory examinations revealed the following: a high white blood cell count of 11.87×10^9/L (reference range: 3.50-9.50×10^9/L); low hemoglobin of 117 g/L (reference ranage: 130–175 g/L); and low serum sodium of 132 mmol/L (reference range: 137–147 mmol/L). High-sensitivity C-reactive protein, procalcitonin and eight infectious disease markers showed no significant abnormalities. A cranial CT scan revealed no significant abnormalities. A chest CT scan indicated mild chronic inflammation in both lungs. Ultrasonography of the urinary system showed prostatic hyperplasia with calcification.

The patient received anti-infection treatment involving moxifloxacin (0.4 g q.d. for five days), imipenem-cilastatin (1 g q.i.d. for five days) and doxycycline (0.1 g q.d. for one day), but the condition progressively worsened with recurrent fever. On 5 March 2026, the patient developed clouded consciousness, disorientation, impaired calculation ability and difficulty urinating and defecating. The patient’s family requested a transfer to another hospital.

On 10 March 2026, the patient was admitted to a different tertiary medical center in China. A neurological examination revealed nuchal rigidity and muscle strength of grade 5 in all four limbs, as well as negative pathological signs. Examination of muscle tone and coordination was unsuccessful. The CASE score was 13 (memory dysfunction: 3 points; psychiatric symptoms: 3 points; language dysfunction: 3 points; gait instability: 3 points; fatigue: 1 point), the MRS score was 4, the bladder function score was 4, and the rectal function score was 3.

Laboratory examinations: a high white blood cell count of 9.59×10^9/L (reference range: 3.50-9.50×10^9/L), low hemoglobin of 128 g/L (reference range: 130–175 g/L), low serum sodium of 131.4 mmol/L (reference range: 137–147 mmol/L), low serum creatinine of 51.1 umol/L (reference range: 57–111 umol/L). Interleukin detection, rheumatic three items, ENA profile, anti-neutrophil cytoplasmic antibodies, glycosylated hemoglobin, and tumor three items showed no abnormalities.

PET-CT revealed diffuse gaseous distension of the ileocecum, colon and rectum. There were no definite signs of hypermetabolic malignant tumours throughout the body.

**Figure 2:**
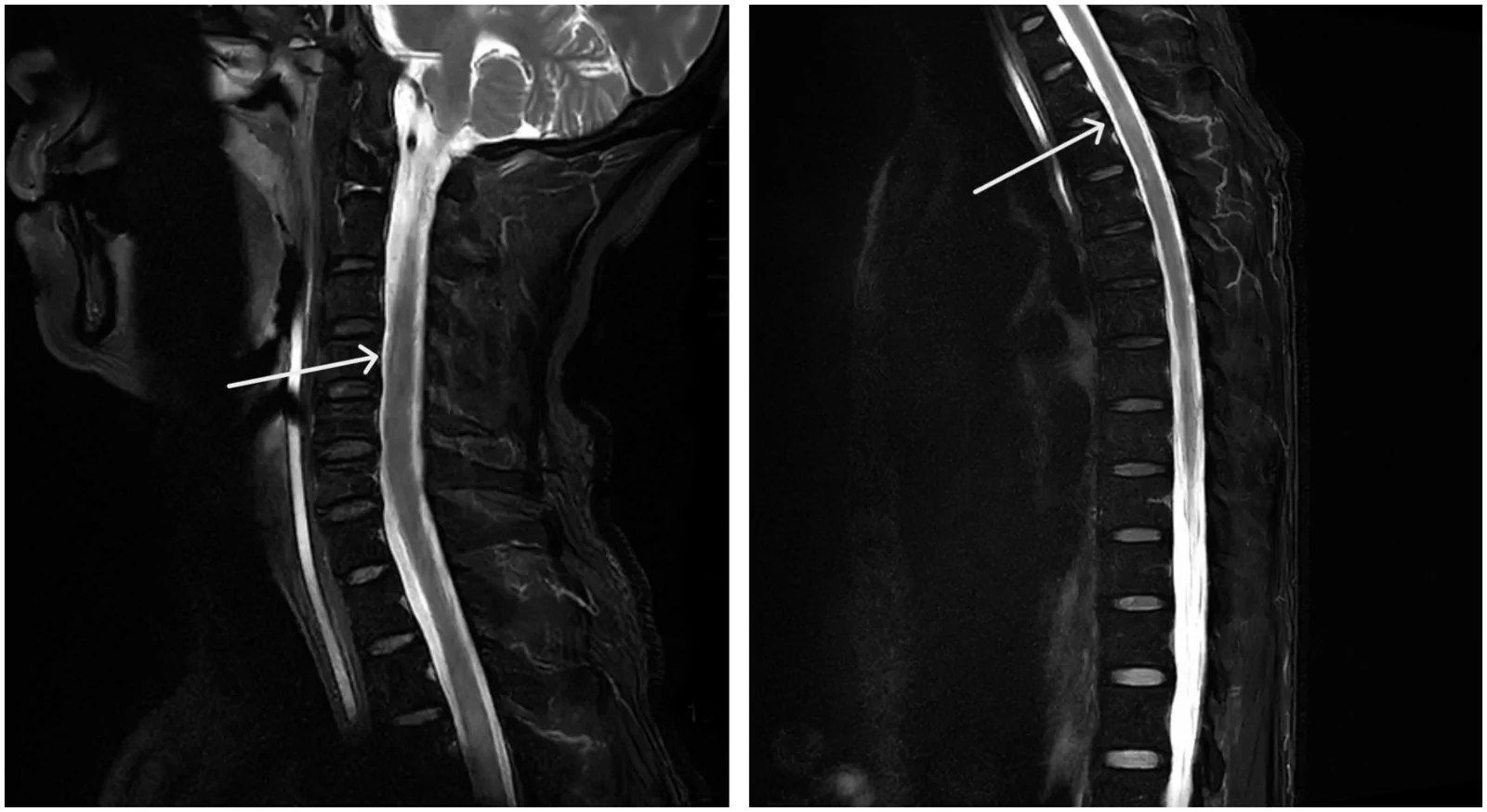
Magnetic resonance imaging of the cervical and thoracic spine of patient 3. T2-weighted imaging shows hyperintensity in the C2, C4, C5, and T2-T4 vertebral body levels, suggesting inflammatory lesions.

Cranial MRI (plain and enhanced) showed a few ischemic foci in the bilateral basal ganglia and thalamus. Cervical + thoracic + lumbar spine MRI (plain + enhanced) showed intramedullary lesions at the C2, C4, C5, and T2-T4 levels, considered inflammatory lesions (**Figure 2**). CSF examination showed: pressure 230 mmH2O (reference range 80–180 mmH2O), white blood cell count 120×10^6/L (reference range 0-8×10^6/L), glucose 2.78 mmol/L (reference range 2.8-4.5 mmol/L), chloride 106.3 mmol/L (reference range 120–132 mmol/L), IgG content 0.151 g/L (reference range 0-0.1 g/L). CSF protein quantitative analysis showed 1.02 g/L (reference range 0.15-0.45 g/L). CSF pathogen and microbial detection indicated suspected Enterococcus cecum infection. CSF GFAP antibody IgG positive (1:100).

The patient was diagnosed with GFAP-A and received intravenous methylprednisolone pulse therapy (500 mg per day) from 14 March 2026 onwards. On 20 March 2026, the treatment was switched to oral methylprednisolone tablets (24 mg once daily). The patient’s level of consciousness and fatigue improved, with no significant change in urinary or defecation dysfunction.

Admitted to our hospital on 24 March 2026. Neurological physical examination: The patient was conscious and gave coherent responses, but speech was slightly slurred. Both pupils were equal in size and round, with sensitive light reflexes, and the eyeballs moved freely, with no nystagmus. Bilateral nasolabial grooves were symmetrical, the tongue was midline on protrusion. Muscle strength in all four limbs was grade 5-. Limb muscle tone was normal. Bilateral sensory function was symmetrical. Bilateral finger-to-nose tests were accurate. Romberg sign was positive. The neck was supple and the bilateral Babinski signs were negative. The CASE score was 5 (language dysfunction: 1 point; gait instability: 3 points; fatigue: 1 point), the mRS score was 4, the bladder function score was 4 and the rectal function score was 3.

The patient was diagnosed with GFAP-A and was receiving maintenance treatment with methylprednisolone tablets (24 mg, once daily). However, significant abdominal distension and severe difficulty urinating and defecating persisted. After thorough communication with the patient’s relatives, we administered intravenous efgartigimod at a dose of 800 mg on the 2nd day of admission. The patient reported relieved fatigue, regained the ability of independent ambulation, and presented with significant remission of abdominal distension. When the urinary catheter was clamped, the patient experienced a sensation of needing to urinate, but was still unable to urinate independently. On the 6th day of admission, efgartigimod was administered again at a dose of 800 mg intravenously. By the time of discharge, the patient had no abdominal distension and could urinate and defecate independently. The CASE score was 1 (gait instability: 1 point), the mRS score was 1, the bladder function score was 1 and the rectal function score was 0.

## Discussion

GFAP-A is a rare central nervous system autoimmune disease distinct from neuromyelitis optica spectrum disorder and multiple sclerosis. GFAP-A typically presents with an acute or subacute onset, and its etiology remains unclear. It may be associated with viral infections (e.g., Epstein-Barr virus and herpes simplex virus), tumors, or autoimmune abnormalities; viral infections are the most common trigger. GFAP is an intermediate filament protein expressed in astrocytes that is involved in various biological functions. This disease predominantly affects the meninges, brain parenchyma, spinal cord, and optic nerves. Common clinical manifestations include meningitis, encephalitis, myelitis, visual abnormalities and autonomic dysfunction (5).

The majority of studies indicate that cranial MRI scans reveal abnormalities in over 80% of patients. MRI findings of cerebral lesions can affect multiple areas, including the frontal, parietal, temporal and occipital lobe; the insula; the internal capsule; the thalamus; the basal ganglia; the corpus callosum; the cerebral peduncle; and the brainstem. These lesions typically manifest as punctate, patchy, or confluent abnormal signals. They appear as low signal intensity on T1-weighted imaging, high signal intensity on T2-weighted imaging and fluid-attenuated inversion recovery (FLAIR) sequences and isointensity on diffusion-weighted imaging (DWI). Spinal MRI demonstrates abnormalities in over 50% of patients, commonly affecting the cervical or thoracic spinal cord in long segments, with associated enhancement of the central canal or meninges (18). The characteristic imaging finding of GFAP-A is linear enhancement radiating from the periventricular white matter into the adjacent white matter (19).

The brain MRIs of the 3 patients in this article did not show typical GFAP-A manifestations. The spinal MRI of case 3 showed lesions at the C2, C4, C5, and T2-T4 levels, involving the cervical and thoracic segments with extensive involvement (spanning multiple vertebral segments), which partially aligns with the “long segment involvement” characteristic of GFAP encephalitis. However, this patient lacked central/peripheral distribution and enhancement characteristics, and “T2 high signal” alone could not fully match the typical pattern of GFAP encephalitis. The imaging manifestations of GFAP-A are highly heterogeneous, and GFAP-A affects different parts of the central nervous system (brain, spinal cord, optic nerve) and different layers (meninges, perivascular, brain parenchyma, spinal cord center), inevitably leading to variations in imaging presentation. Typical manifestations (such as periventricular radial perivascular enhancement) do not occur in all patients; some patients may only present with leptomeningeal enhancement, isolated myelopathy, or extensive white matter lesions, which falls within the natural spectrum of the disease. The disease cannot be definitively excluded or confirmed solely based on the “presence of typical imaging findings”. A comprehensive diagnosis requires consideration of disease heterogeneity, lesion stage, comorbidities, and imaging limitations (20). GFAP antibodies are specific biomarkers for this disease. The diagnosis of GFAP-A primarily relies on laboratory detection of GFAP antibodies in serum and cerebrospinal fluid, with cerebrospinal fluid GFAP antibody being the gold standard. Antibody titer is an important reference indicator for evaluating the condition of GFAP-A, but it is not an absolute positive correlation and requires comprehensive judgment in combination with clinical practice (1, 21). For patients with a negative cranial MRI scan, a diagnosis of GFAP-A is primarily based on positive cerebrospinal fluid (CSF) GFAP antibodies, coupled with clinical manifestations consistent with GFAP-A.

Due to the relative rarity and complex, diverse clinical manifestations of GFAP-A, established and unified treatment guidelines are currently lacking. Based on clinical practice and existing research, the first-line treatment for GFAP-A in the acute phase typically involves high-dose corticosteroid pulse therapy followed by sequential treatment. For patients who do not respond well to hormone therapy, are severe ill, or have other co-existing autoimmune antibodies, the combined use of IVIG or plasma exchange can also improve clinical symptoms (22). However, some patients respond poorly to hormone therapy, and the relapse rate during hormone dose reduction is relatively high, posing significant challenges for clinical treatment. A retrospective cohort study of 233 patients with GFAP-A showed that around 25% had an unfavorable prognosis (modified Rankin Scale score≥3) (23) and did not respond well to hormone therapy.

Autonomic dysfunction is a common and indicative clinical manifestation of GFAP-A. However, we often focus on recovering higher neural function disorders while neglecting autonomic dysfunction. Autonomic dysfunction includes gastrointestinal dysfunction, bladder dysfunction, heart rate variability, blood pressure variability, and abnormal sweating (24, 25). Bladder dysfunction primarily manifests as difficulty urinating, increased urinary frequency, urge incontinence, and overflow incontinence. Gastrointestinal dysfunction may present with symptoms such as abdominal pain, diarrhea, abdominal distension, and hiccups (26, 27). Autonomic dysfunction is most commonly characterized by bladder dysfunction and intestinal paralysis. The incidence of autonomic dysfunction in GFAP-A patients is high. The most common neurological sequelae during follow-up for patients with corticosteroid insensitivity, recurrence, or refractory disease are cognitive dysfunction, followed by urinary and defecatory dysfunction (4).

All three of the patients reported in this article presented with prodromal symptoms of an upper respiratory tract infection. These included cognitive impairment of varying degrees, psychiatric symptoms, neurological deficits and autonomic dysfunction, which was primarily characterized by urinary and defecatory disorders. Combined with positive cerebrospinal fluid GFAP antibody IgG, GFAP-A can be definitively diagnosed. All 3 GFAP-A patients exhibited significant autonomic dysfunction. As the patients refused IgG treatment and our hospital lacked the conditions for plasmapheresis, this posed challenges for subsequent treatment.

The pathogenesis of GFAP-A is primarily attributed to abnormal immune system responses triggered by GFAP antigens. These responses involve a complex interplay of factors, including antigen exposure, immune activation, antibody-mediated inflammation, CD8+ T cell-mediated cellular immunity and blood-brain barrier disruption. Ultimately, this leads to astrocytic damage, neuroinflammation, and neurological dysfunction. Pathogenic antibodies (anti-GFAP IgG) are a central driver of disease activity (1, 12). Efgartigimod rapidly, significantly, and reversibly reduces serum total IgG and specific autoantibody levels by blocking the FcRn-IgG interaction, thereby preventing the circulation of endogenous IgG (including pathogenic autoantibodies) and accelerating their catabolism (28). Recently, cases (16, 29) have been reported indicating that for GFAP-A patients unresponsive to hormone therapy, efgartigimod can rapidly reduce pathogenic antibody levels, achieving swift clinical and imaging (lesion absorption) improvements, thereby offering a novel targeted therapeutic strategy for hormone-insensitive GFAP-A.

All 3 patients with GFAP-A reported herein presented with autonomic dysfunction characterized by urination and defecation disorders. Patient 1 exhibited symptoms of severe urinary retention and paralytic ileus; following treatment with a high-dose of corticosteroids combined with efgartigimod, their urination and defecation functions rapidly improved. Patient 2 presented with autonomic dysfunction manifesting as urinary incontinence and difficulty defecating. Their autonomic dysfunction showed no significant improvement following treatment with low-dose corticosteroids. Following treatment with efgartigimod, the patient’s urination and defecation functions had largely returned to normal by the time of discharge. Patient 3 continued to exhibit significant autonomic dysfunction, presenting as urinary retention and paralytic ileus, even after treatment with high-dose corticosteroids. After treatment with efgartigimod, their autonomic dysfunction improved markedly. Improvements in other clinical symptoms were also observed in all 3 patients after they were treated with efgartigimod (**Table 1**). None of the patients experienced any adverse events related to the drug during treatment with efgartigimod or during the four-month follow-up period, indicating a favorable safety profile in this patient population.

**Table 1:** Changes in clinical scale scores before and after efgartigimod treatment in the 3 patients.

Clinical scale	Timing	Case1	Case2	Case3
**CASE** score	**before treatment**	10	4	5
	**after treatment**	1	0	1
**mRS** score	**before treatment**	4	2	4
	**after treatment**	1	1	1
bladder function score	**before treatment**	4	2	4
	**after treatment**	1	0	1
Rectal function score	**before treatment**	3	2	3
	**after treatment**	1	0	0

CASE (Clinical Assessment Scale in Autoimmune Encephalitis) , mRS (Modified Rankin Scale).

In our cases, after combination therapy with efgartigimod and corticosteroids, significant improvement in autonomic dysfunction was observed in all 3 patients (**Tables 2**–**4**). The standard recommended dose of efgartigimod is typically 10 mg/kg weekly. Pharmacodynamic studies from the phase I clinical trial of efgartigimod indicated that doses of 10 to 25 mg/kg administered every 4–7 days did not result in cumulative toxicity and were considered safe (13). Studies (15, 30) have used a higher dose of efgartigimod combined with a special dosing regimen (administering 20 mg/kg efgartigimod on day 1 and day 5) to treat autoimmune diseases, which provides us with another treatment approach. The dose of efgartigimod used for Case 1 and Case 2 in this article was 20 mg/kg. Initially, we recommended a dose of 20 mg/kg for Case 3; however, due to he patient’s financial constraints, we opted for a dose of 15 mg/kg. Case 2 received only 1 injection of efgartigimod, and the patient showed significant improvement in clinical symptoms. Considering the patient’s financial situation, a second dose was not administered.

**Table 2:** Timeline of diagnosis and treatment for case 1.

Date of admission	Event	Treatment
the 1st day	The patient had fever and cough, accompanied by mental abnormalities, and difficulty with urination and defecation.	The patient was treated with cephalosporins.
the 5th day	The patient still had fever, significant restlessness and delirium, accompanied by abdominal distension and urinary and bowel dysfunction.	The patient was treated with piperacillin-tazobactam, ganciclovir, and 10 mg dexamethasone.
the 7th day	The patient’s fever peak had decreased, and restlessness had slightly improved, but the urinary and bowel dysfunction and incoherent speech remain significant.	The patient received 10 mg of dexamethasone and continued the original anti-infective treatment regimen.
the 9th day	The patient had been diagnosed with GFAP-A.	The patient received 1 g methylprednisolone pulse treatment.
the 11th day	The patient’s delirium and restlessness had improved, and the abdominal distension had improved, but the urinary and bowel dysfunction remained obvious.	The patient received 1200mg of efgartigimod treatment.
the 12th day	The patient’s symptoms of irritability and incoherent speech had resolved, abdominal distension had improved, and urinary and bowel function had returned to normal.	The patient’s corticosteroid dose was reduced, and 500mg methylprednisolone was administered.
the 15th day	The patient was conscious and not delirious, but still had urinary and bowel dysfunction.	The patient received 1200mg of efgartigimod treatment.
the 20th day	The patient was able to void spontaneously and pass stool independently.	Discharge

**Table 3:** Timeline of diagnosis and treatment for case 2.

Date	Event	Treatment
10 days before admission	The patient experienced chills and fever	The patient took cephalosporins
5 days before admission	The patient had no fever but developed constipation, frequent urination, urgency, and urinary incontinence.	The patient did not receive treatment
Day 1 of admission	The patient developed dizziness, fatigue with delayed reaction, slurred speech, and urinary and bowel dysfunction.	The patient received treatment with ceftriaxone, ganciclovir, and 10 mg dexamethasone.
Day 4 of admission	The patient had been diagnosed with GFAP-A.	The patient received 1200mg efgartigimod combined with 20mg dexamethasone.
Day 7 of admission	The patient’s dizziness and fatigue had improved, the symptoms of slow reaction and slurred speech had disappeared, and the bowel and bladder function had improved.	The patient continued to receive 20mg dexamethasone and the original anti-infective treatment regimen.
Day 10 of admission	The patient experienced occasional dizziness, had normal muscle strength in the limbs, and had normal bowel and bladder function.	Discharge

**Table 4:** Timeline of diagnosis and treatment for case 3.

Date of admission	Event	Treatment
the 1st day	The patient presented with slurred speech, unsteady gait, and fatigue, accompanied by abdominal distension and severe urinary and bowel dysfunction.	The patient received treatment with methylprednisolone tablets (24mg, once daily).
the 2nd day	The patient had been diagnosed with GFAP-A and presented with significant abdominal distension and severe urinary and bowel dysfunction.	The patient received 800mg of efgartigimod treatment.
the 3rd day	The patient’s speech was improved, gait was improved, and fatigue was improved. Abdominal distension was significantly reduced, but urinary and bowel function showed no significant improvement.	The patient continued to use methylprednisolone tablets (24mg, once daily).
the 6th day	The patient had mild gait instability, but symptoms of slurred speech and fatigue had resolved. There was no abdominal distension, and bowel function had improved, although urinary dysfunction remained significant.	The patient received 800mg of efgartigimod treatment.
the 9th day	The patient’s bowel function was normal, and urinary function had improved.	Discharge

How efgartigimod alleviates neuroinflammation controlling bladder and bowel function, with possible potential mechanisms as follows: GFAP-IgG antibodies are the core drivers of GFAP-A neuroinflammation. Abnormal antibodies bind to astrocytes, triggering cytotoxic T cell and B cell-mediated immune attacks, leading to inflammatory damage of neural tissues, including neural regions controlling autonomic functions. Efgartigimod rapidly clears GFAP-IgG antibodies, directly cutting off the source of antibody-mediated immune attacks, reducing inflammatory infiltration and destruction of neural tissues, thereby alleviating the dysfunction of bladder and bowel control caused by neuroinflammation (31). Efgartigimod’s FcRn blockade effect is not limited to reducing antibody titers; it also affects antigen presentation, modulates T cell subset function, and promotes regulatory plasma cell function. By restoring local immune homeostasis in the nervous system, efgartigimod helps to mitigate the continuous progression of neuroinflammation, promote the repair of damaged neural functions, and consequently improve the neuroregulatory functions of the bladder and bowel (32).

This article primarily aimed to evaluate the efficacy of Efgartigimod in improving autonomic function. However, it must be noted that patients received concurrent baseline immunosuppressive therapy with corticosteroids during efgartigimod treatment. This combination therapy inevitably introduced significant confounding factors, primarily manifested in three aspects: Firstly, corticosteroids possess a clear effect on improving neurological function with a delayed therapeutic onset, making it difficult to distinguish their immediate effects from those of efgartigimod in the short term. Secondly, some patients experienced spontaneous disease remission within the timeframe of combination therapy, further obscuring the true drug effects. Thirdly, there might be synergistic immunosuppressive effects between efgartigimod and corticosteroids, leading to additive or interactive impacts on autonomic function improvement. Given that this study is a retrospective case series lacking a perfectly parallel monotherapy control group, it is impossible to fully correct the aforementioned confounding factors through statistical methods, nor to isolate the independent therapeutic effect of efgartigimod. Future validation through prospective, randomized controlled trials is necessary.

The limitations of this article are as follows: 1. As a single-center retrospective analysis, due to the rarity of the disease, the sample size is relatively small, limited by the scope of clinical medical record data collection. 2. Patients were treated with efgartigimod in combination with corticosteroids, making it difficult to completely isolate the effects of the combination therapy. The independent efficacy of efgartigimod may introduce certain confounding bias into the efficacy evaluation. 3. The dosage of efgartigimod used in this study was not completely unified, which make it difficult to draw general or regular conclusions about the relationship between dose differences and clinical outcomes. 4. Patients had low compliance, and dynamic evolution data of cerebrospinal fluid GFAP antibody titers and serum GFAP-IgG levels could not be obtained, thus failing to deeply explore the dynamic correlation between antibody level changes and clinical efficacy. 5. When evaluating the bladder and rectal function of patients, this study mainly used applying simplified urinary and rectal scoring systems for quantitative assessment, and did not adopt internationally recognized standardized autonomic nervous system assessment tools (e.g., COMPASS scale, heart rate variability detection, sudomotor function testing). This was mainly because this study is a single-center retrospective study, limited by clinical practical conditions, sample size, and assessment costs, it is difficult to carry out time-consuming and equipment-intensive standardized autonomic function tests comprehensively in routine clinical practice. Simplified clinical scoring is simple to operate and easy to promote in clinical practice, which can better reflect the overall degree of impairment of bladder and rectal function in patients and provide basic data for the clinical analysis of this study. However, simplified scoring has certain limitations in assessing subtle differences in autonomic function and objective quantification. Future large-sample, multi-center studies combined with standardized objective assessment tools will help to further verify and improve the relevant clinical assessment systems. 6. The follow-up time of this study was relatively short, and the long-term efficacy and long-term safety of efgartigimod could not be fully tracked and evaluated. This case report mainly found that the combination of efgartigimod and corticosteroid treatment can significantly improve corticosteroid-refractory autonomic dysfunction in GFAP-A patients, and future multi-center randomized controlled trials (RCTs) are needed for further verification.

## Conclusion

We observed significant remission of corticosteroid-refractory autonomic dysfunction in GFAP-A patients following combination therapy with efgartigimod and corticosteroids. Despite several limitations, efgartigimod may serve as an alternative treatment option for patients with GFAP-A and corticosteroid-insensitive, refractory autonomic dysfunction, warranting further validation through large-scale cohort studies and retrospective analyses in the future.

## Data Availability

The original contributions presented in the study are included in the article/supplementary material. Further inquiries can be directed to the corresponding authors.
